# Expression of CD24 in Human Bone Marrow-Derived Mesenchymal Stromal Cells Is Regulated by TGF*β*3 and Induces a Myofibroblast-Like Genotype

**DOI:** 10.1155/2016/1319578

**Published:** 2015-12-14

**Authors:** Luisa Marilena Schäck, Manuela Buettner, Alexander Wirth, Claudia Neunaber, Christian Krettek, Andrea Hoffmann, Sandra Noack

**Affiliations:** ^1^Trauma Department, Hannover Medical School, 30625 Hannover, Germany; ^2^Institute of Laboratory Animal Science, Hannover Medical School, 30625 Hannover, Germany; ^3^Cellular Neurophysiology, Hannover Medical School, 30625 Hannover, Germany

## Abstract

Human bone marrow-derived stromal cells (hBMSCs) derived from the adult organism hold great promise for diverse settings in regenerative medicine. Therefore a more complete understanding of hBMSC biology to fully exploit the cells' potential for clinical settings is important. The protein CD24 has been reported to be involved in a diverse range of processes such as cancer, adaptive immunity, inflammation, and autoimmune diseases in other cell types. Its expression in hBMSCs, which has not yet been analyzed, may add an important aspect in the understanding of hBMSC biology. The present study therefore analyzes the expression, regulation, and functional implication of the surface protein CD24 in hBMSCs. Methods used are stimulation studies with TGF beta as well as shRNA-mediated knockdown and overexpression of CD24 followed by microarray, immunocytochemistry, and flow cytometric analyses. To our knowledge, we demonstrate for the first time that the expression of CD24 is an inherent property of hBMSCs. Importantly, the data links the upregulation of CD24 to the adoption of a myofibroblast-like gene expression pattern in hBMSCs. We demonstrate that CD24 is an important modulator in transforming growth factor beta 3 (TGF*β*3) signaling with a reciprocal regulatory relationship between these two proteins.

## 1. Introduction

Human bone marrow-derived stromal cells (hBMSCs) are the* in vitro* progeny of human mesenchymal stem cells (hMSCs) found in the bone marrow. It has been demonstrated that hBMSCs can contribute to the healing process of kidney, lung, cardiac, and liver damage via both the release of paracrine factors and differentiation [1–4]. hBMSCs are also able to develop into myofibroblast-like cells and thereby play a beneficial role in physiological tissue remodeling. However, this property can also play a detrimental role in pathological processes. For example, excessive fibrotic growth can cause further damage to organs and carcinoma-associated fibroblasts derived from hMSCs can sustain cancer progression [5–12]. Therefore, it is of importance to understand the underlying mechanisms driving hBMSCs to adopt a myofibroblast-like phenotype.

The glycoprotein CD24 is expressed by a great variety of different cell types, such as epithelial cells, hepatocyte progenitor cells, embryonic stem cells, and certain hematopoietic cells and also in certain myofibroblast cells and has accordingly been implied to play a role in diverse biological processes [13–16]. For example, CD24 has been shown to serve in the discrimination between pathogen-associated molecular patterns and danger-associated molecular patterns in dendritic cells [17]. It is known to play a role in the migration of certain cancer cells and has been shown to be upregulated in regenerating tissue in inflammatory bowel disease [18–20]. However, a possible function for CD24 in hBMSCs has so far not been analyzed. The present study therefore serves to elucidate the expression and functional implication of CD24 in hBMSCs.

## 2. Materials and Methods

### 2.1. Purification and Culture of hBMSCs

For studies involving human tissues, we obtained ethical approval from the ethical committee of the Medical School Hannover. Written informed consent was obtained from all donors. All personal information was made anonymous. Bone marrow aspirates were harvested by iliac crest aspiration during routine orthopedic procedures. hBMSCs were isolated from fresh bone marrow aspirates by density gradient centrifugation and subsequent plastic adhesion of mononuclear cells as described elsewhere [21]. Cells were cultured at 37°C with 5% CO_2_ at 85% humidity in MSC growth medium (DMEM FG 0415 (Biochrom, Berlin, Germany) with 10% (v/v) FBS (not heat-inactivated) (Thermo Fisher Scientific, Schwerte, Germany), 20 mM HEPES, 1% (100 U/mL/100 *μ*g/mL) penicillin/streptomycin (Biochrom AG, Berlin, Germany), and 2 ng/mL human recombinant FGF2 (PeproTech, Hamburg, Germany)). The cells were passaged at a density of around 70% with 0.025% Trypsin-EDTA solution and seeded at a density of 2 × 10^3^ cells per cm^2^. Alternative culture medium to verify whether CD24 expression was not an artifact induced by FGF-2 or FBS consisted of alpha-medium (F0925, Biochrom), 10% AB-Human Serum (Life Technologies, Carlsbad, California, USA), 1% (100 U/mL/100 *μ*g/mL) penicillin/streptomycin, and 1% glutamine [22]. Cells were routinely analyzed for their expression of CD29, CD44, CD73, CD90, and CD105 and differentiation potential into the adipogenic, chondrogenic, and osteogenic lineage as described before [23]. The age and gender of the donors whose hBMSCs were used for microarray analyses are listed in Supplemental Table 1 in Supplementary Material available online at http://dx.doi.org/10.1155/2016/1319578.

### 2.2. Flow Cytometry

hBMSCs were detached and washed with FACS buffer (2% (v/v) FBS in phosphate-buffered saline (PBS)) (×2). All centrifugation steps were performed at 300 ×g and 4°C for 5 minutes. For each sample, 1 × 10^5^ cells were used. For intracellular analysis, cells were fixed and permeabilized: cells were centrifuged and resuspended in PBS supplemented with 0.5% (w/v) paraformaldehyde and 0.01% (w/v) Tween-20. After incubation (20 minutes, 37°C, and shaking at 400 rpm) and washing with FACS buffer (×2), cells were resuspended in PBS supplemented with 0.5% (w/v) saponin and 1% (w/v) BSA (Saponin buffer) and incubated for 10 minutes at room temperature and 400 rpm.

For both intra- and extracellular analyses, cells were then centrifuged and incubated with appropriate fluorochrome-conjugated antibodies from BioLegend (San Diego, USA) for 30 minutes at 4°C in the dark. After washing with FACS buffer (×2) (intracellular staining: Saponin buffer) cells were analyzed using a FACS Canto (BD Biosciences, Franklin Lakes, USA) recording 1 × 10^4^ cells. Dead cells were excluded by using scatter parameters using BD FACSDiva Software and Flowing Software version 2.5.0.

### 2.3. Stimulation of hBMSCs

hBMSCs were stimulated with 10 ng/mL TGF*β*3 for 7 days. As controls, cells were treated with TGF*β*3 in combination with either the chemical SB431542 (20 mM; Merck Millipore, Schwalbach, Germany), which can be used to block Alk5 receptor-dependent TGF beta signaling, or a neutralizing anti-TGF*β*3 antibody (4 *μ*g/mL; R&D Systems, Minneapolis, USA).

### 2.4. Immunocytochemistry

hBMSCs were grown on poly-L-lysine coated cover slips (15 × 15 mm) in 24-well plates and fixed with 4% (w/v) paraformaldehyde in PBS. After washing with PBS (×3), the cells were blocked and permeabilized (5% (w/v) BSA, 0.5% (w/v) Triton X-100 in PBS) for 60 minutes at room temperature and then washed again with PBS (×3). Appropriate primary antibodies (monoclonal mouse anti-CD24 antibody (ML5) (BioLegend), polyclonal rabbit anti-CD24 antibody (FL-80, Santa Cruz Biotechnology, Dallas, USA), and monoclonal mouse anti-alpha smooth muscle actin (R&D Systems, Minneapolis, Minnesota, USA)) were used at a concentration of 1 *μ*g/mL in 1% (w/v) BSA and 0.1% (w/v) Triton X-100 in PBS and incubated for 1 hour at room temperature. After washing with 0.1% (w/v) Triton X-100 in PBS (×3), the cells were incubated with Alexa Fluor 488 or Alexa Fluor 568 anti-mouse antibody (Life Technologies) (2 *μ*g/mL in 1% (w/v) BSA, 0.1% (w/v) Triton X-100 in PBS) for 1 h at room temperature in the dark. Cells were washed with PBS (×3), mounted on microscope slides using Immunoselect Antifading Mounting Medium with 4′,6-diamidino-2-phenylindole (DAPI) (Dianova GmbH, Hamburg, Germany), and subsequently analyzed using a BZ-9000 Generation II Fluorescence Microscope (Keyence, Neu-Isenburg, Germany). The z-stack image was captured with a Zeiss LSM 780 (Carl Zeiss AG, Oberkochen, Germany).

### 2.5. RNA Preparation, cDNA Synthesis, and Quantitative Real Time (qRT) PCR Analysis

RNA preparation and cDNA synthesis were performed as described before [23]. RNA concentration and purity were determined by measuring the absorption at 260/280 nm. cDNA synthesis of 1 *μ*g RNA was carried out with random hexamer primers (RevertAid First Strand cDNA-Synthesis Kit, Thermo Fisher Scientific) after performing DNase treatment (Thermo Fisher Scientific) to remove genomic DNA according to the protocol of the supplier. qRT-PCR analysis was used to quantify the transcripts of CD24 (Hs02379687_s1), TGF*β*3 (Hs01086000_m1), COL1A1 (Hs00164004_m1), IDO1 (Hs00984148_m1), TNAP (Hs01029144_m1), and ACTG2 (Hs01123712_m1). 18S-rRNA (Hs9999901_s1) served as housekeeping gene. The gene specific assays and the Fast Advanced Mastermix were purchased from Life Technologies.

### 2.6. CD24 Overexpression and Knockdown

CD24 was cloned from cDNA generated from induced pluripotent stem cells with a forward primer containing a BamH I and a reverse primer containing a Sal I restriction site (forward 5′-TATAGGATCCGCCACCATGGGCAGAGCAATGGTGGCCAGG-3′, reverse 5′-TATAGTCGACTTATTAAGAGTAGAGATGCAGAAAGAGAGAG-3′). The cDNA was amplified using Pfu Ultra DNA-polymerase (Agilent Technologies, Böblingen, Germany), purified in an agarose gel and extracted from the gel using the GeneJet Gel-Extraction Kit (Thermo Fisher Scientific) according to the instructions of the manufacturer. The lentiviral expression vector pLOX/TW, which is under control of a tetracycline-responsive CMV promoter, as well as the cloned CD24 construct were restriction digested with Sal I and BamH I. The plasmid was subsequently treated with shrimp alkaline phosphatase (Affymetrix, Santa Clara, USA) according to the instructions of the manufacturer. After ligation of the CD24 construct into the pLOX/TW-vector, the vector was transformed into* E. coli SURE* bacteria by electroporation. Plasmid preparation of pLOX/TW/CD24 was performed using JETSTAR Plasmid Kits (Genomed, Löhne, Germany) according to the instructions of the manufacturer. The integrity of the construct was confirmed by sequencing using the ABI Prism 310 capillary sequencer (BigDye Terminator Cycle Sequencing Ready Reaction Mix version 1.1; Applied Biosystems, Darmstadt, Germany). The lentiviruses used for transduction of all plasmids were produced as described before [24]. Lentivirus-containing supernatants were harvested and filtered 48 hours and 72 hours after transfection and were combined. In order to use similar amounts of viral RNA copy numbers the virus titer was determined using the NucleoSpin RNA Virus Kit (Macherey-Nagel, Düren, Germany) and the Lenti-X qRT-PCR Titration Kit (Clontech Laboratories, Mountain View, CA, USA) according to the instructions of the manufacturers.

shRNA directed against CD24 (GIPZ Lentiviral Human shRNA Gene Set (glycerol set) CD24) and a scrambled control shRNA were purchased from Thermo Fisher Scientific and prepared according to the instructions of the manufacturer. Plasmid preparation was performed using JETSTAR Plasmid Kits (Genomed) according to the instructions of the manufacturer. The integrity of the construct was confirmed by sequencing using the ABI Prism 3. After lentiviral transduction the cells were selected by Puromycin (20 *μ*g/mL for 5 days).

### 2.7. Western Blotting

Western blotting was performed as described before [25]. Briefly, proteins were extracted using lysis buffer (5 mL 1 M Tris Base pH 7.5, 37.5 mL 1 M NaCl, 2.5 mL 1 M KCl, 2.5 mL 0.2 M EDTA pH 7.5, 2.5 g Igepal CA-630, 0.5249 g NaF, and 0.1115 g Na_4_P_2_O_7_  × H_2_O containing proteinase inhibitor). The fractionation was performed by using 10% sodium dodecyl sulfate-polyacrylamide gel electrophoresis. The proteins were then transferred to a polyvinylidene fluoride (PVDF) membrane and after blocking with 0.1% fat milk for 30 minutes incubated with the primary antibody against CD24 (polyclonal rabbit anti-CD24 antibody (FL-80), 1 : 2000, Santa Cruz Biotechnology, Dallas, USA), *β*-actin (mouse anti-*β*-actin antibody (AC-15), 1 : 10.000 Sigma Aldrich, St. Louis, USA), or Histone H3 (rabbit anti-Histone H3 antibody, 1 : 1000, BioLegend). The bound primary antibodies were detected using horseradish peroxidase conjugated secondary antibodies and Amersham ECL or Amersham ECL Plus Western Blotting Detection Reagent (GE Healthcare Life Sciences, Chalfont St Giles, Buckinghamshire, Great Britain). Films were developed using CAWOMAT 2000IR (CAWO, Schrobenhausen, Germany).

### 2.8. Transcriptome Analysis

The Whole Human Genome Oligo Microarray Kit 4x44K v2 (G4845A, design ID 026652, Agilent Technologies) was utilized in this study. Microarrays of this design type contain 44495 probes, covering roughly 26000 transcripts. Synthesis of Cy3- or Cy5-labeled cRNA was performed with the “Quick Amp Labeling kit, two-color” (#5190-0444, Agilent Technologies) according to the manufacturer's recommendations. cRNA fragmentation, hybridization, and washing steps were carried out exactly as recommended in the “Two-Color Microarray-Based Gene Expression Analysis Protocol V5.7” (Agilent). Slides were scanned on the Agilent Micro Array Scanner G2565CA (pixel resolution 5 *μ*m, bit depth 20). Data extraction and processing of raw fluorescence intensity values were performed with the “Feature Extraction Software V10.7.3.1” by using the recommended default extraction protocol file: GE2_107_Sep09.xml.

For interarray normalization, processed intensity values as generated in the Feature Extraction process, “gProcessedSignal” (gPS) and “rProcessedSignal” (rPS), were further transformed as follows: all PS values (gPS and rPS) of one particular microarray (“Array *i*” in the formula shown below) were multiplied with a microarray-specific scaling factor. This factor was calculated by dividing a “reference 75th Percentile value” (set as 1500 for the whole series) by the 75th Percentile value of the gPS values calculated by the Feature Extraction software for that microarray. Accordingly, normalized PS values for all samples (microarray data sets) were calculated by the following formula:

(1) normalized  PSArray  i=PSArray  i×150075th  Percentile  gPSArray  i.

The data of the samples from the overexpression of CD24 were further processed as follows: a lower intensity threshold was defined as 1% of the reference 75th Percentile value (=15). All of those normalized PS values that fell below this intensity border were substituted by the respective surrogate value of 15.

Ingenuity Pathway Analysis 5 (IPA) by Ingenuity Systems (Redwood City, California, USA) was used to analyze the transcriptome data. For CD24 knockdown and TGF*β*3 stimulation two independent biological replicates were analyzed. For CD24 overexpression one biological sample was used and analyzed twice as a technical replicate. For the analysis, the threshold for genes to be considered as significantly regulated was set at 1.5. The IPA option Canonical Pathway Analysis was used to identify canonical pathways that were significant to the microarray data input. The significance of the association between the data set and the canonical pathway was determined based on two parameters: (1) a ratio of the number of genes from the data set that map to the pathway divided by the total number of genes that map to the canonical pathway and (2) a *P* value calculated using Fischer's exact test determining the probability that the association between the genes in the data set and the canonical pathway is due to chance alone [26].

### 2.9. Statistical Analyses

Statistical analyses were performed using Tukey-Kramer Test in Prism 5.0 (GraphPad Software Inc., San Diego, USA). *P* values are indicated as ^*∗*^ *P* ≤ 0.05, ^*∗∗*^ *P* ≤ 0.01, and ^*∗∗∗*^ *P* ≤ 0.001.

## 3. Results

### 3.1. CD24 Is Expressed in hBMSCs

**Figure 1:**
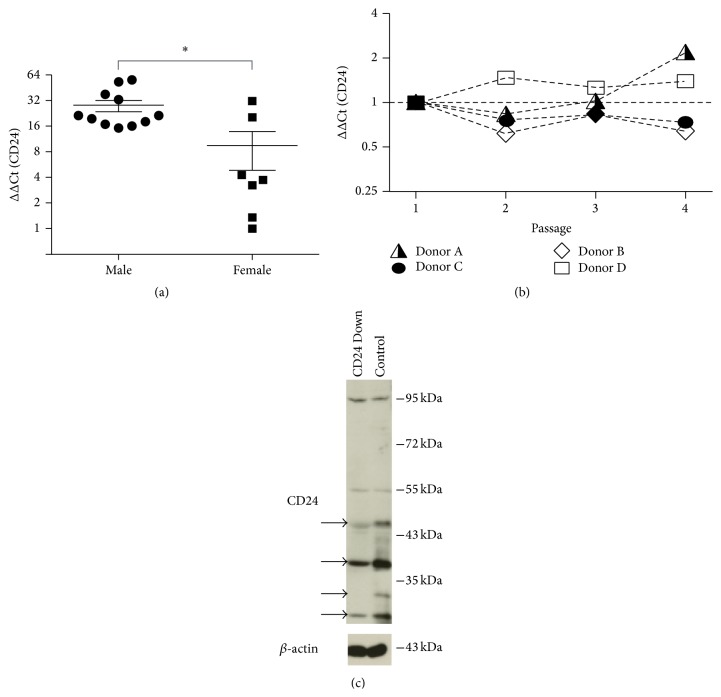
Analysis of CD24 expression in hBMSCs. (a) hBMSC mRNA from 18 different donors in passage 2 of* in vitro* culture were analyzed for the expression of CD24 mRNA by qRT-PCR and analyzed for gender-dependent differences in mRNA expression levels. The relative expression values are plotted as fold change in mRNA expression (ΔΔCt) relative to the sample with the lowest relative CD24 mRNA expression. (b) The mRNA expression of CD24 was analyzed from passage 1 to passage 4 of* in vitro* culture in 4 individual donors. The expression values are normalized to the expression levels of the respective donor in passage 1 (ΔΔCt). (c) The expression of CD24 protein was evaluated by western blot with a polyclonal rabbit anti-CD24 antibody. In order to determine which bands were specific for CD24 hBMSCs were transduced with either a shRNA directed against CD24 mRNA (CD24 Down) or a scrambled shRNA (Control). Cell extracts from these two groups were then compared for CD24 reactive bands: the western blot bands with reduced intensity in the CD24 Down Group are marked with black arrows and represent bands specific for CD24. *β*-actin was used as loading control.

hBMSC populations from 18 different donors were analyzed for the expression of CD24 mRNA in passage 2 of* in vitro* culture by qRT-PCR. All populations tested were positive for the mRNA of CD24 with hBMSCs from male donors containing significantly higher relative CD24 mRNA levels than hBMSCs from female donors (Figure 1(a)). The mRNA expression of CD24 was found to be stable during the culture* in vitro* as analyzed for the first four passages (Figure 1(b)).

The expression of CD24 on protein level was confirmed by western blot. Several positive bands corresponding to CD24 variants described in the literature were found [16]. In order to assess the specificity of these bands, hBMSCs were lentivirally transduced with a vector containing a short hairpin RNA (shRNA) directed against the CD24 mRNA. The resulting knockdown of CD24 led to a reduction of CD24 protein expression enabling the identification of bands specific for CD24 (marked by arrows in Figure 1(c)). *β*-actin was used as loading control.

### 3.2. Cellular Localisation of CD24 Protein

**Figure 2:**
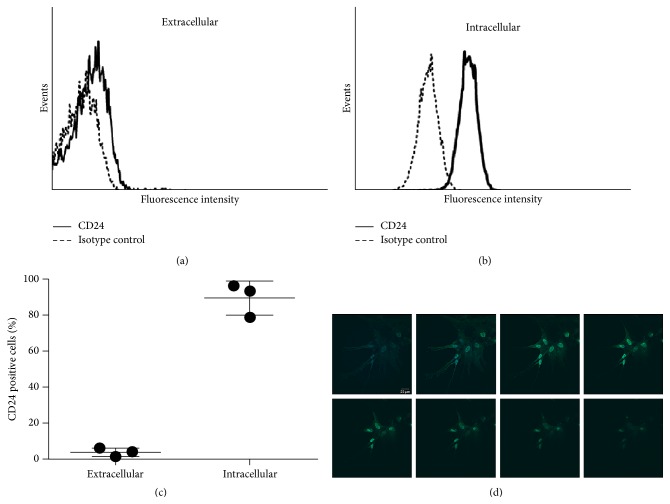
Cellular localisation of CD24. (a), (b), and (c) hBMSCs were analyzed by flow cytometry for the expression of CD24 protein. An exemplary histogram depicting the low extracellular CD24 expression can be found in (a); an exemplary histogram depicting the high intracellular CD24 protein density is shown in (b). (c) shows the percent expression of CD24 protein extra- and intracellularly in three independent experiments with hBMSCs from different donors. (d) Immunocytochemical analysis of the intracellular CD24 protein expression showing a diffuse cytosolic and a strong nuclear reactivity. A z-stack image is shown to highlight the nuclear localisation of CD24 (monoclonal anti-CD24 antibody, clone ML-5). Green = CD24. Blue = nuclear staining. Scale: 20 *μ*m. A high resolution image of (d) can be found in Supplemental Figure 5.

Flow cytometric analyses of CD24 in hBMSCs showed that CD24 was expressed on the cell surface of hBMSCs at a very low density (Figure 2(a)). In contrast to this, its expression was found at a high density intracellularly (Figure 2(a)). Furthermore, intracellular immunocytochemistry revealed not only a diffuse cytosolic staining pattern but also a nuclear staining (Figure 2(b)). Strikingly, the nuclear reactivity was present not only when staining with a polyclonal antibody but also when using a monoclonal antibody generated against the protein core of CD24 (clone ML-5) (Supplemental Figure 1).

In order to exclude the possibility that the observed expression of CD24 could be an artifact induced by the use of basic fibroblast growth factor (FGF2) or fetal bovine serum (FBS) in the cell culture medium, hBMSCs were cultured not only in hBMSC medium supplemented with 2 ng/mL human FGF2 and 10% fetal bovine serum (FBS) but also in hBMSC-AB medium that contained neither FBS nor FGF2 and was supplemented with 10% human AB serum instead (hBMSC-AB medium). The expression of CD24 was similar in both media as evaluated by immunocytochemistry and flow cytometry (Supplemental Figure 2).

### 3.3. Reciprocal Regulation of CD24 and TGF*β*3

**Figure 3:**
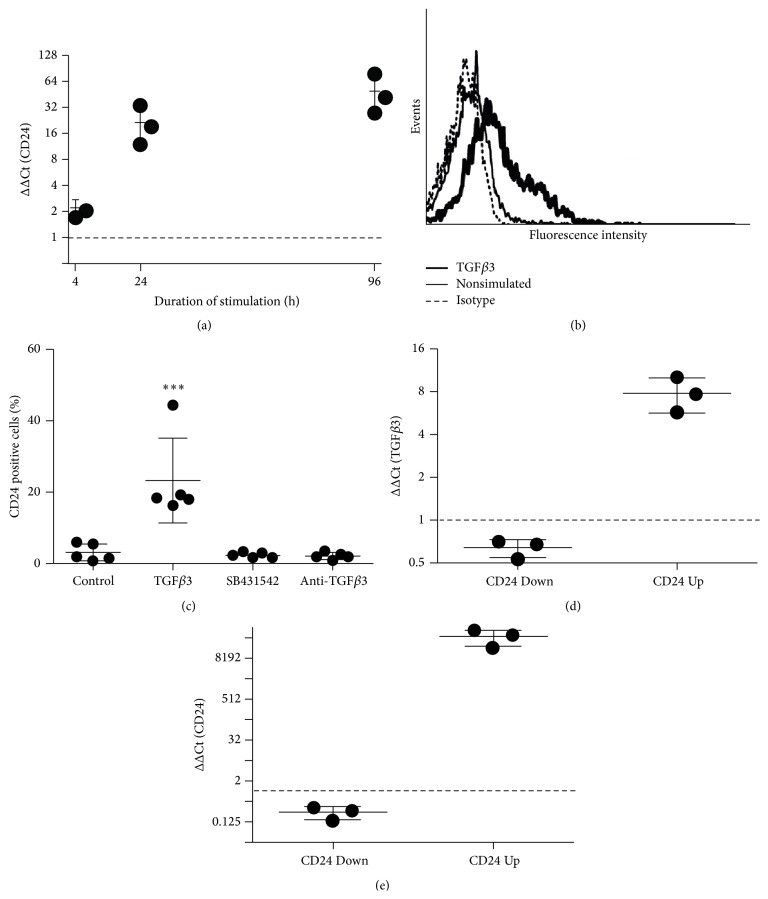
Reciprocal regulation of CD24 and TGF*β*3. (a) qRT-PCR analysis of CD24 mRNA expression after stimulation with 10 ng/mL TGF*β*3 for 4 h, 24 h, and 96 h relative to unstimulated control cells (ΔΔCt). (b) Exemplary histogram showing induction of CD24 surface expression after treatment with 10 ng/mL TGF*β*3 or with 2.5 ng/mL TGF*β*1 for 7 days as analyzed by flow cytometry. (c) Flow cytometric analysis of CD24 surface protein expression on hBMSCs after 7 days in unstimulated control cells (Control), in cells treated with 10 ng/mL TGF*β*3 (TGF*β*3), in cells treated with 10 ng/mL TGF*β*3 + 20 *μ*M SB431542 (SB431542), or in cells treated with 10 ng/mL TGF*β*3 and 4 *μ*g/mL anti-TGF*β*3 antibody (anti-TGF*β*3). (d) and (e) qRT-PCR analysis of TGF*β*3 (d) and CD24 (e) mRNA expression after knockdown (CD24 Down) or overexpression (CD24 Up) of CD24 in hBMSCs after 7 days relative to respective control hBMSCs (*n* = 3 independent experiments with hBMSCs from different donors).

The stimulation of hBMSCs with 10 ng/mL of TGF*β*3 resulted in an average 22.0- (±11.1-) fold increase in CD24 mRNA 24 hours after stimulation which was increased to 50.1- (±26.5-) fold after 96 hours of stimulation relative to unstimulated control cells (Figure 3(a)). Moreover, stimulation of hBMSCs with TGF*β*3 for 7 days also led to an increase in CD24 surface protein expression (Figure 3(b)). This effect demonstrated here with the TGF*β*3 isoform was not limited to TGF*β*3 but was achievable as well by stimulating hBMSCs with TGF*β*1 (data not shown), which may be explained by the fact that TGF beta isoforms show partly overlapping functions when supplied as active forms* in vitro* [27]. As evaluated by flow cytometry, the mean CD24 surface protein expression rose to 23.3% (±11.9%) after stimulation with TGF*β*3 with control cells showing a mean CD24 surface expression of 3.0% (±2.4%) (Figure 3(c)). The stimulatory effect of TGF*β*3 on the surface expression of CD24 was blockable both by use of a neutralizing antibody directed against TGF*β*3 and by the Alk4, Alk5, and Alk7 inhibitor SB431542 (Figure 3(c)). In contrast to TGF*β*, stimulation with BMP2 had no significant influence on the expression of CD24 on mRNA or protein level (data not shown). Interestingly, the knockdown of CD24 via shRNA (CD24 Down) led to a reduced expression of TGF*β*3, and overexpression of CD24 by lentiviral transduction (CD24 Up) resulted in an elevated TGF*β*3 mRNA expression (Figure 3(d)). Overexpression and knockdown of CD24 in these samples were verified by qRT-PCR (Figure 3(e)).

### 3.4. Transcriptome Analysis of hBMSCs after Overexpression or Knockdown of CD24

In order to elucidate possible biological functions of CD24 in hBMSCs transcriptome analyses of hBMSCs overexpressing CD24 (CD24 Up Group), of hBMSCs with shRNA-mediated knockdown of CD24 expression (CD24 Down Group), and of hBMSCs stimulated with 10 ng/mL TGF*β*3 (TGF*β*3 Group) were compared to transcriptome data of respective control hBMSCs. The qRT-PCR analysis of CD24 mRNA expression in the samples used for transcriptome analysis can be found in Supplemental Figure 3. The number of genes significantly regulated after overexpression of CD24, after knockdown of CD24, or after stimulation with TGF*β*3 is listed in Supplemental Table 2: overall, stimulation with TGF*β*3 had the strongest effect with 3615 genes up- or downregulated. CD24 overexpression led to the regulation of 528 and CD24 knockdown to the regulation of 1132 genes (cutoff 1.5). The top 10 up- and downregulated genes for each of the three groups are listed in Supplemental Table 3. The comparison of genes regulated in the CD24 Up Group and in the CD24 Down Group showed that 51 genes were regulated in opposing directions between these two groups (cutoff 1.5) (Supplemental Table 4). Exemplary genes were analyzed by qRT-PCR to verify the microarray data (Supplemental Figure 4).

Analysis of upstream regulators after knockdown or overexpression of CD24 revealed that CD24 may play a role in TGF beta signaling in hBMSCs (Table 1): the activity of TGF*β*1 or the inactivity of the inhibitory Smad, Smad7, was identified as possible causative agents of the observed changes in gene expression after overexpression of CD24. Conversely, the changes in gene expression after knockdown of CD24 were predicted to be caused by the inactivity of TGF*β*1 signaling or the activity of Smad7.

**Figure 4:**
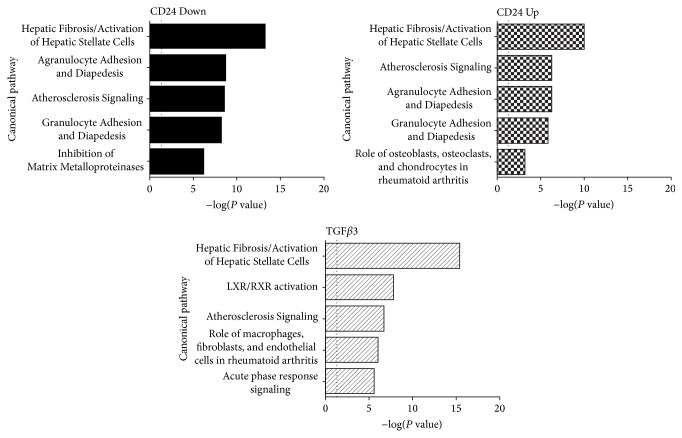
Biological functions influenced by CD24 and TGF*β*3. The top five canonical pathways regulated by knockdown of CD24 (CD24 Down), overexpression of CD24 (CD24 Up), or stimulation with TGF*β*3 (TGF*β*3) in hBMSCs based on their significance (*P* value) calculated by Ingenuity Pathway Analysis using the right-tailed Fisher's Exact Test using the entire data set. The dotted line is the threshold value with *P* = 0.05.

IPA was used to analyze canonical pathways that were significantly influenced by the knockdown of CD24, by the overexpression of CD24, or by the stimulation of hBMSCs with TGF*β*3. In Figure 4 the top five canonical pathways for each of these three treatment groups are listed based on their significance (*P* value). In Supplemental Table 5 the values of all three groups for all canonical pathways represented in Figure 4 can be found. Four of the signaling pathways that ranked in the top five pathways of the CD24 Up Group were also listed in the top five canonical pathways of the CD24 Down Group:* Hepatic Fibrosis/Activation of Hepatic Stellate Cells*,* Agranulocyte Adhesion and Diapedesis*,* Atherosclerosis Signaling*, and* Granulocyte Adhesion and Diapedesis*. In all three groups these four pathways were significant with a *P* value below 0.05 (Supplemental Table 5).

### 3.5. CD24 Expression Correlates to the Expression of Myofibroblast-Like Marker Genes

**Figure 5:**
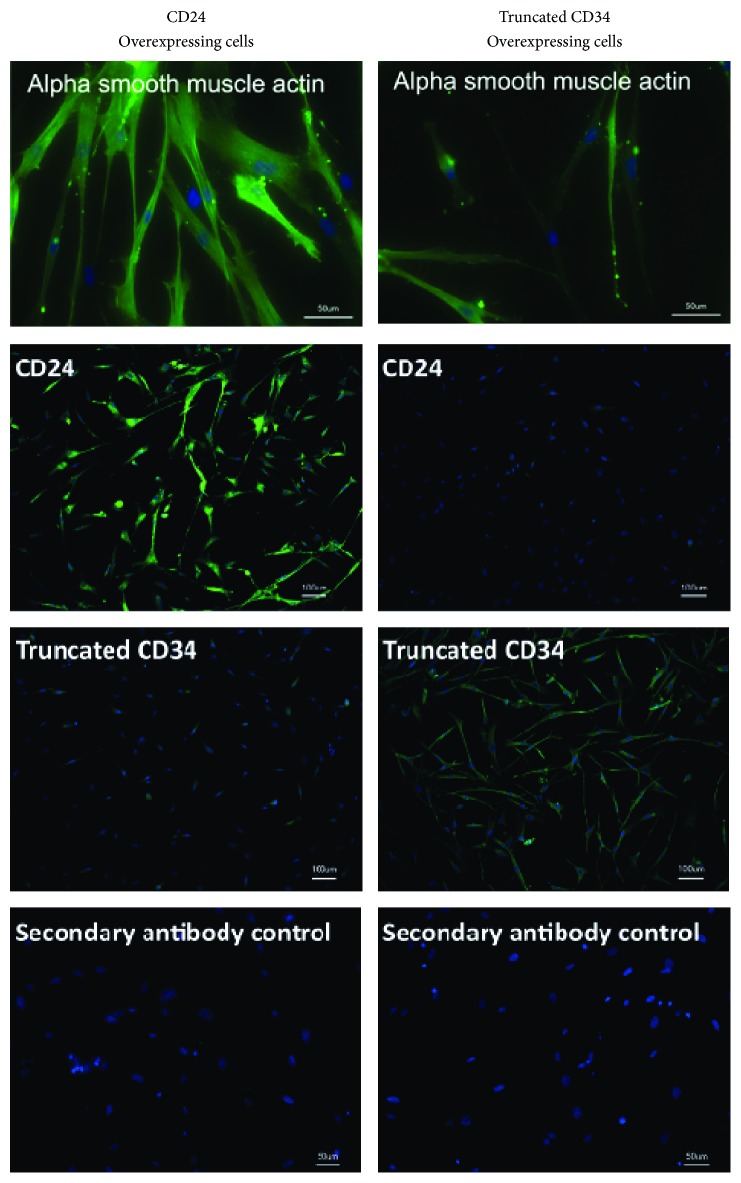
Expression of alpha smooth muscle actin. Intracellular immunocytochemical staining of alpha smooth muscle actin after overexpression of CD24 (CD24, left column) or truncated CD34 as control (CD34, right column). Alpha smooth muscle actin: staining of alpha smooth muscle actin (green), and nuclear staining (blue) show that in hBMSCs overexpressing CD24 reactivity for alpha smooth muscle actin increases relative to control cells (Scale 50 *μ*m). CD24: staining of CD24 (green), and nuclear staining (blue) verify that transduction rate of CD24 is nearly complete (Scale 100 *μ*m). CD34: staining of CD34 (green), and nuclear staining (blue) verify that control cells were transduced efficiently with truncated CD34 (Scale 100 *μ*m). Secondary antibody control: staining control with secondary antibody only (green) and nuclear staining (blue) (Scale 50 *μ*m).

The canonical pathway* Hepatic Fibrosis/Activation of Hepatic Stellate Cells* was the pathway most significantly represented in all three groups (CD24 Up, CD24 Down, and TGF*β*3). Myofibroblasts play an important role in the pathogenesis of Hepatic Fibrosis. In accordance with the representation of this pathway the myofibroblast marker genes ACTA2, CNN1, and COL1A1 were significantly regulated in all three groups (Supplemental Table 6). In order to analyze whether the upregulation of myofibroblast-marker gene ACTA2 after overexpression of CD24 would also result in an enhanced expression of its protein product (alpha smooth muscle actin), hBMSCs overexpressing CD24 or control hBMSCs (hBMSCs overexpressing a truncated form of CD34 that contains only six intracellular amino acids and therefore lacks any signaling domains) were analyzed by immunocytochemical staining. The overexpression of CD24 led to an increase in expression of alpha smooth muscle actin as compared to the control (Figure 5). Overexpression of CD24 and truncated CD34 was verified by staining of CD24 and CD34.

## 4. Discussion

CD24 is a highly glycosylated protein that has been described in many different cell types. It is often associated with a pluripotent cell state and has subsequently been used as a negative marker for the selection of hBMSCs generated from pluripotent stem cells (iPSCs) using flow cytometry [28, 29]. Only recently, a study found CD24 to be expressed in mesenchymal stem cell-enriched cell populations, both on mRNA by qRT-PCR and on protein level by protein array [30]. The present study was conducted to analyze the expression and cellular localization of CD24 in hBMSCs and to gain insight into the functional relevance of this protein in hBMSCs. The results presented here establish that CD24 is present in human bone marrow-derived stromal cells both on mRNA and on protein level irrespective of cell culture medium supplements such as FGF2 and fetal bovine serum. qRT-PCR analysis of CD24 mRNA in passage 2 of* in vitro* culture in hBMSC samples from 18 different donors revealed up to 60-fold differences in relative mRNA expression levels with cells from male donors expressing significantly higher levels of CD24 mRNA compared to cells from female donors. It is known that the expression of CD24 can be downregulated by estrogens and upregulated by androgens which could explain the observed differences between the relative expression levels of CD24 mRNA in hBMSCs from female and male donors [31, 32]. Under normal cell culture conditions the CD24 protein was only expressed at a low density on the cell surface of hBMSCs and was found predominantly intracellularly, which may explain the (flow cytometry-based) assumption that the protein is not expressed by hBMSCs [33]. Intracellular immunocytochemical staining revealed that the reactivity for the CD24 protein was not restricted to the cytosol but was also found in the cell nuclei as analyzed by confocal microscopy. The specificity of immunocytochemical CD24 staining in the nuclei was validated both by staining with a second, monoclonal antibody directed against the protein CD24 and by western blot using nuclear cell fractions. The localisation of CD24 has been described predominantly on the cell surface of different cell types whereas a nuclear localisation for CD24 has been described only once before: Ahmed et al. used immunohistochemical analyses and found CD24 to be expressed in the cell nuclei of neoplastic epithelial cells but not in cell nuclei of normal epithelial cells or of nonepithelial cells [34]. CD24 can interact with nucleolin, a ubiquitous protein that can function as a nuclear shuttle protein opening the possibility that nucleolin might facilitate the nuclear import of CD24 in hBMSCs [17]. Further studies are needed to elucidate both the mechanism by which CD24 can be transferred to the cell nucleus of hBMSCs and the function of CD24 in a nuclear context.

Interestingly, whereas the basal level of CD24 expression at the cell membrane was relatively low, stimulation with either TGF*β*3 or TGF*β*1 led to an increase in CD24 expression at the cell membrane. The stimulatory action of TGF*β* species on CD24 cell surface expression in hBMSCs seems all the more interesting as a reciprocal relationship between CD24 and TGF*β*3 was found to exist* in vitro*: CD24 was inducible by TGF*β*3 stimulation and TGF*β*3 was inducible by CD24 overexpression. Accordingly, knockdown of CD24 expression by shRNA led to a reduction in the relative mRNA expression levels of TGF*β*3. This close relationship between these two molecules was underlined by the changes in the gene expression pattern in hBMSCs both after knockdown and overexpression of CD24: analysis with the software IPA revealed that the influence exerted by the overexpression of CD24 on the transcriptome of hBMSCs closely resembled the effect that stimulation with TGF*β*1 could have. Conversely, the influence exerted by the knockdown of CD24 on the transcriptome of hBMSCs was predicted to possibly stem from inhibiting TGF*β*1 signaling. As would be expected this close regulatory relationship seems to also be reflected in shared biological functionality: the canonical pathway most significantly affected in all three groups was* Hepatic Fibrosis/Activation of Hepatic Stellate Cells*. Notably the top five canonical pathways of the CD24 Down group were also significantly regulated in CD24 Up as well as TGF*β*3 group (although not necessarily among the top five most significant canonical pathways). The same is true for the top five canonical pathways of the CD24 Up group as well as the TGF*β*3 group compared to the other groups respectively. This raises the possibility that CD24 is an integral part of TGF beta signaling in hBMSCs.

Hepatic Fibrosis is a wound healing response that depends on the Activation of Hepatic Stellate Cells (HSCs). These HSCs are perivascular mesenchymal cells that upon liver injury are activated into proliferative, fibrogenic, and contractile myofibroblasts [35]. It is known that upon TGF beta stimulation hBMSCs adopt a myofibroblast-like phenotype* in vitro* and that mesenchymal stem cells express myofibroblast marker genes such as alpha smooth muscle actin (ACTA2) upon engraftment to wounds* in vivo* [36–40]. The overexpression of CD24 in hBMSCs seemed to induce a similar process, whereas the knockdown of CD24 seemed to exert a converse reaction: the mRNA of the fibrotic markers alpha smooth muscle actin and collagen Ia1 were upregulated in the CD24 Up but downregulated in the CD24 Down Group. Furthermore, the transcription factor Hairy/Enhancer-of-Split related to YRPW Motif 2 (HEY2) that is known as an important regulator of vascular smooth muscle cell accumulation during vascular remodeling was also upregulated in the CD24 Up and downregulated in the CD24 Down Group [41]. The upregulation of forkhead box S1 (FOXS1) in the CD24 Up Group and its downregulation in the CD24 Down Group were also in line with the formation of myofibroblast-like cells as blood vessel-associated smooth muscle cells and pericytes in the central nervous system stain positive for FOXS1 [42]. Finally, the induction of chordin-like 2 (CHRDL2), an inhibitor of BMP signals, further underlined the profibrotic activity of CD24 because certain BMP species exert antifibrotic effects [43]. Thus, the upregulation of CD24 seemed to favor the development of a myofibroblast-like genotype whereas the downregulation of CD24 seemed to inhibit the expression of myofibroblast marker genes in hBMSCs. The genotype may be reflected by the acquisition of an according phenotype as the overexpression of CD24 in hBMSCs resulted in an increased expression of alpha smooth muscle protein. The fact that myofibroblasts express CD24 is also in line with these results [13, 44].

The representation of both “*Agranulocyte Adhesion and Diapedesis*” and “*Granulocyte Adhesion and Diapedesis*” processes among the top 5 significant canonical pathways in both the CD24 Up and the CD24 Down Group and among the significant pathways in the TGF*β*3 Group was striking and may signify that apart from playing a role in the induction of a myofibroblast-like phenotype CD24, like TGF beta, it also functions in the regulation of immunomodulatory processes in hBMSCs. It has been documented that hepatic myofibroblasts attract lymphocytes through the release of chemokines and cytokines [45–47]. Smooth muscle cells that are closely related to myofibroblasts also play a key role in the pathogenesis of atherosclerosis, where they might dedifferentiate into myofibroblast-like cells, which could explain why “*Atherosclerosis Signaling*” is found among the relevant canonical pathways in both the CD24 Up and the CD24 Down Group [48]. Myofibroblasts are known to modulate the extracellular matrix via both metalloproteinases and tissue inhibitors of metalloproteinases, which may explain why the knockdown of CD24 in hBMSCs led to changes in mRNA expression associated with the canonical pathway “*Inhibition of Matrix Metalloproteinases*” [49, 50].

## 5. Conclusion

The data presented in this study demonstrates that CD24 is expressed in hBMSCs irrespective of donor, culture duration, and the medium supplements FBS and FGF2. Furthermore, we show that a reciprocal regulatory relationship exists between CD24 and TGF*β*3 and more importantly that modulation of CD24 expression influences the gene expression pattern of hBMSCs in a similar fashion as does stimulation with TGF*β*3 which suggests that CD24 may play a regulatory role in several TGF-beta dependent processes, especially in the development of hBMSCs into myofibroblast-like cells. Therefore, CD24 may present a promising target to inhibit the unwanted development of myofibroblasts or even myofibroblast cell-like carcinoma-associated fibroblasts from hBMSCs. Future studies in immune-deficient mice are planned to investigate the* in vivo* relevance of the findings described in this paper.

## Supplementary Material

Supplemental Table 1. Gender and age of the donors whose hBMSCs were used for microarray analysis.
Supplemental Table 2. Number of genes regulated by group stimulus in hBMSCs relative to respective control hBMSCs.
Supplemental Table 3. List of top 10 up- and downregulated genes from CD24 Up Group, CD24 Down Group, and TGFβ3 Group
Supplemental Table 4. Genes with opposed expression in the CD24 Down sample and in the CD24 Up sample relative to the respective controls (fold induction ≥ 1.5). CD24 is not found in this list because the sequence of CD24 detected by the microarray probe lies in the non-coding region of CD24. CD24 was cloned from the CD24 cDNA, therefore the non-coding region was not cloned. The up- and downregulation of CD24 was instead verified by qRT-PCR using a probe with a target sequence in the coding region of CD24 (see Supplemental Figure 3).
Supplemental Table 5. Complete –log(p-value) values of the top 5 canonical pathways of CD24 Down, CD24 Up and TGFβ3 for all three groups as determined by Ingenuity Pathway Analysis.
Supplemental Table 6. Microarray data of myofibroblast-marker genes significantly regulated in all three microarray groups (CD24 Up, CD24 Down, and TGFβ3).
Supplemental Figure 1. Immunocytochemical staining of CD24 with two different antibodies. Staining of hBMSCs with either a monoclonal anti-CD24 antibody (clone ML-5) or a polyclonal anti-CD24 antibody (both 2 µg/ml) after fixation and permeabilization of hBMSCs led to similar staining patterns with a diffuse cytosolic and a strong nuclear reactivity for CD24 (red). Nuclear staining with DAPI is shown in blue.
Supplemental Figure 2. Comparison of CD24 expression between hBMSCs cultivated with hBMSC (FBS and FGF2) and hBMSC-AB (AB-Serum) medium. A. Intracellular immunocytochemical analysis of CD24 expression revealed a similar staining pattern for CD24 in hBMSCs after culture in hBMSC or hBMSC-AB medium. Monoclonal anti-CD24 antibody is shown in green, and DAPI staining is shown in blue. Scale: 20 µm. B. Flow cytometric analysis of intracellular CD24 expression revealed that CD24 expression is not caused by either FBS or FGF2.
Supplemental Figure 3. qRT-PCR analysis of CD24 mRNA expression after knockdown of CD24, after overexpression of CD24,) or after stimulation with 10 ng/ml TGFβ3. mRNA expression changes relative to the respective controls were as follows: CD24 knockdown led to a 0.30 fold induction (± 0.06 , n = 2 biological replicates, measured with 2 technical replicates each), CD24 overexpression led to a 31065.11 fold induction (n = 1 biological replicate, measured with 2 technical replicates), and TGFb3 led to a 16.29 fold induction (± 4.01 , n = 2 biological replicates, measured with 2 technical replicates each).
Supplemental Figure 4. qRT-PCR validation of microarray expression data of CD24 Up and CD24 Down Group relative to respective controls.
Supplemental Figure 5. This is the high resolution image of Figure 2D.

## Figures and Tables

**Table 1:** IPA upstream regulator analysis. Molecules resulting in similar changes in the pattern of gene expression as the overexpression of CD24 or the knockdown of CD24 are designated with high activational *z*-score values. Listed are those molecules with a *z*-score higher than 2 while exhibiting opposing algebraic signs in the CD24 Up and CD24 Down Groups.

Upstream regulators	Activational *z*-score		Alias	Function of encoded protein
	CD24 Up	CD24 Down		
SMAD7	−3,435	2,136	MAD (Mothers Against Decapentaplegic, Drosophila) Homolog 7	Antagonist of signaling by TGF*β*1 receptor superfamily members [23]

NF-*κ*B1	−2,995	2,222	NF-*κ*B1	Transcription factor that plays a key role in regulating many intracellular signaling events and the immune response to infection [24]

IFNAR1	−2,566	2,927	Interferon (alpha, beta, and omega) receptor 1	Forms one of the two chains of a receptor for interferons alpha and beta [25]

Y 27632	−2,558	2,198		Inhibitor of p160 rho-associated protein kinase (ROCK) [26]

CR1L	−2,412	2,728	Complement component (3b/4b) receptor 1-like	May function as membrane-bound inhibitor of complement activation [27]

NPR1	−2,345	2,426	Natriuretic peptide receptor A/guanylate cyclase A	Receptor for certain natriuretic peptides [28]

SRF	2,404	−2,257	Serum response factor	Transcription factor that binds to the serum response element [29]
