# Supplementary material for: Expression of CD24 in Human Bone Marrow-Derived Mesenchymal Stromal Cells Is Regulated by TGFβ3 and Induces a Myofibroblast-Like Genotype
Source: Stem Cells Int. 2015 Dec 14;2016:1319578. doi: 10.1155/2016/1319578 (PMC4691640; doi:10.1155/2016/1319578)
Supplement: Supplementary file 1 — Supplemental Table 1. Gender and age of the donors whose hBMSCs were used for microarray analysis. Supplemental Table 2. Number of genes regulated by group stimulus in hBMSCs relative to respective control hBMSCs. Supplemental Table 3. List of top 10 up- and downregulated genes from CD24 Up Group, CD24 Down Group, and TGFβ3 Group Supplemental Table 4. Genes with opposed expression in the CD24 Down sample and in the CD24 Up sample relative to the respective controls (fold induction ≥ 1.5). CD24 is not found in this list because the sequence of CD24 detected by the microarray probe lies in the non-coding region of CD24. CD24 was cloned from the CD24 cDNA, therefore the non-coding region was not cloned. The up- and downregulation of CD24 was instead verified by qRT-PCR using a probe with a target sequence in the coding region of CD24 (see Supplemental Figure 3). Supplemental Table 5. Complete –log(p-value) values of the top 5 canonical pathways of CD24 Down, CD24 Up and TGFβ3 for all three groups as determined by Ingenuity Pathway Analysis. Supplemental Table 6. Microarray data of myofibroblast-marker genes significantly regulated in all three microarray groups (CD24 Up, CD24 Down, and TGFβ3). Supplemental Figure 1. Immunocytochemical staining of CD24 with two different antibodies. Staining of hBMSCs with either a monoclonal anti-CD24 antibody (clone ML-5) or a polyclonal anti-CD24 antibody (both 2 µg/ml) after fixation and permeabilization of hBMSCs led to similar staining patterns with a diffuse cytosolic and a strong nuclear reactivity for CD24 (red). Nuclear staining with DAPI is shown in blue. Supplemental Figure 2. Comparison of CD24 expression between hBMSCs cultivated with hBMSC (FBS and FGF2) and hBMSC-AB (AB-Serum) medium. A. Intracellular immunocytochemical analysis of CD24 expression revealed a similar staining pattern for CD24 in hBMSCs after culture in hBMSC or hBMSC-AB medium. Monoclonal anti-CD24 antibody is shown in green, and DAPI staining is shown in [file 1319578.f1.docx]

**Supplemental Table and Figures:**

**Supplemental Table and Figure Legends**

**Supplemental Table 1.** Gender and age of the donors whose hBMSCs were used for microarray analysis.

**Supplemental Table 2.** Number of genes regulated by group stimulus in hBMSCs relative to respective control hBMSCs.

**Supplemental Table 3.** List of top 10 up- and downregulated genes from CD24 Up Group, CD24 Down Group, and TGFβ3 Group.

**Supplemental Table 4.** Genes with opposed expression in the CD24 Down sample and in the CD24 Up sample relative to the respective controls (fold induction ≥ 1.5). CD24 is not found in this list because the sequence of CD24 detected by the microarray probe lies in the non-coding region of CD24. CD24 was cloned from the CD24 cDNA, therefore the non-coding region was not cloned. The up- and downregulation of CD24 was instead verified by qRT-PCR using a probe with a target sequence in the coding region of CD24 (see Supplemental Figure 3).

Supplemental Table 5. Complete –log(p-value) values of the top 5 canonical pathways of CD24 Down, CD24 Up and TGFβ3 for all three groups as determined by Ingenuity Pathway Analysis.

Supplemental Table 6. Microarray data of myofibroblast-marker genes significantly regulated in all three microarray groups (CD24 Up, CD24 Down, and TGFβ3).

Supplemental Figure 1. Immunocytochemical staining of CD24 with two different antibodies. Staining of hBMSCs with either a monoclonal anti-CD24 antibody (clone ML-5) or a polyclonal anti-CD24 antibody (both 2 µg/ml) after fixation and permeabilization of hBMSCs led to similar staining patterns with a diffuse cytosolic and a strong nuclear reactivity for CD24 (red). Nuclear staining with DAPI is shown in blue.

Supplemental Figure 2. Comparison of CD24 expression between hBMSCs cultivated with hBMSC (FBS and FGF2) and hBMSC-AB (AB-Serum) medium. A. Intracellular immunocytochemical analysis of CD24 expression revealed a similar staining pattern for CD24 in hBMSCs after culture in hBMSC or hBMSC-AB medium. Monoclonal anti-CD24 antibody is shown in green, and DAPI staining is shown in blue. Scale: 20 µm. B. Flow cytometric analysis of intracellular CD24 expression revealed that CD24 expression is not caused by either FBS or FGF2.

Supplemental Figure 3. qRT-PCR analysis of CD24 mRNA expression after knockdown of CD24, after overexpression of CD24,) or after stimulation with 10 ng/ml TGFβ3. mRNA expression changes relative to the respective controls were as follows: CD24 knockdown led to a 0.30 fold induction (± 0.06 , n = 2 biological replicates, measured with 2 technical replicates each), CD24 overexpression led to a 31065.11 fold induction (n = 1 biological replicate, measured with 2 technical repliactes), and TGFb3 led to a 16.29 fold induction (± 4.01 , n = 2 biological replicates, measured with 2 technical replicates each).

**Supplemental Figure 4**. qRT-PCR validation of microarray expression data of CD24 Up and CD24 Down Group relative to respective controls.

Supplemental Figure 5. This is the high resolution image of Figure 2D.

**Supplemental Table 1.**

| **Microarray** | **Gender** | **Age** |
| --- | --- | --- |
| CD24 Knockdown 1 / TGF beta 3 stimulation 1 | m | 51 |
| CD24 Knockdown 2 / TGF beta 3 stimulation 2 | m | 28 |
| CD24 overexpression 1 | m | 52 |
| CD24 overexpression 2 | w | 48 |

**Supplemental Table 2.**

| **Group** | **Genes regulated (Cutoff 1.5)** | **Genes downregulated (Cutoff 1.5)** | **Genes upregulated (Cutoff 1.5)** |
| --- | --- | --- | --- |
| CD24 Up | 528 | 195 | 333 |
| CD24 Down | 1132 | 575 | 557 |
| TGFβ3 | 3615 | 1701 | 1914 |

**Supplemental Table 3.**

| **CD24 Up Group** | | | | | | | | | | | | | | | | | | |
| --- | --- | --- | --- | --- | --- | --- | --- | --- | --- | --- | --- | --- | --- | --- | --- | --- | --- | --- |
| **Total number of genes regulated (cutoff 1.5):** | | | | | | | | | 628 | | | | | | | | | |
| **Top 10 upregulated genes** | | | | | | **Top 10 downregulated genes** | | | | | | | | | | | | |
| **Gene** | | **Gene name** | | **mean fold induction** | **SD** | **Gene** | | | | **Gene name** | | | | | **mean fold induction** | | | **SD** |
| **MUC7** | | mucin 7, secreted | | 24.8 | 32.0 | **MMP12** | | | | matrix metallopeptidase 12 (macrophage elastase) | | | | | -5.6 | | | 1.7 |
| **PI15** | | peptidase inhibitor 15 | | 21.4 | 6.4 | **MMP3** | | | | matrix metallopeptidase 3 (stromelysin 1, progelatinase) | | | | | -4.3 | | | 0.7 |
| **AGT** | | angiotensinogen (serpin peptidase inhibitor, clade A, member 8) | | 15.6 | 2.5 | **FOXQ1** | | | | forkhead box Q1 | | | | | -3.4 | | | 1.1 |
| **FOXS1** | | forkhead box S1 | | 12.6 | 6.1 | **IL12A** | | | | interleukin 12A (natural killer cell stimulatory factor 1, cytotoxic lymphocyte maturation factor 1, p35) | | | | | -3.2 | | | 0.5 |
| **CSNK1G1** | | casein kinase 1, gamma 1 | | 10.3 | 2.4 | **RSPO2** | | | | R-spondin 2 homolog (Xenopus laevis) | | | | | -3.2 | | | 0.1 |
| **COL5A3** | | collagen, type V, alpha 3 | | 9.7 | 2.6 | **FAM159B** | | | | SFRS12-interacting protein 1; family with sequence similarity 159, member B | | | | | -3.1 | | | 2.1 |
| **RDH16** | | retinol dehydrogenase 16 (all-trans) | | 9.1 | 1.8 | **MCTP2** | | | | multiple C2 domains, transmembrane 2 | | | | | -2.9 | | | 0.4 |
| **PLXDC1** | | plexin domain containing 1 | | 7.8 | 2.0 | **ABCC9** | | | | ATP-binding cassette, sub-family C (CFTR/MRP), member 9 | | | | | -2.9 | | | 1.2 |
| **LPHN3** | | latrophilin 3 | | 7.7 | 8.5 | **PYHIN1** | | | | pyrin and HIN domain family, member 1 | | | | | -2.9 | | | 0.8 |
| **ANKUB1** | | ankyrin repeat and ubiquitin domain containing 1 | | 6.8 | 1.9 | **SRRM3** | | | | hypothetical protein FLJ37078 | | | | | -2.9 | | | 0.2 |
| **CD24 Down Group** | | | | | | | | | | | | | | | | | | |
| **Total number of genes regulated (cutoff 1.5):** | | | | | | | | 1337 | | | | | | | | | | |
| **Top 10 upregulated genes** | | | | | | | **Top 10 downregulated genes** | | | | | | | | | | | |
| **Gene** | | **Gene name** | **mean fold induction** | | **SD** | | **Gene** | | | | **Gene name** | | | **mean fold induction** | | | **SD** | |
| **CXCL10** | | chemokine (C-X-C motif) ligand 10 | 14.5 | | 14.5 | | **RIMS3** | | | | regulating synaptic membrane exocytosis 3 | | | -12.6 | | | 1.1 | |
| **SLC16A6** | | solute carrier family 16, member 6 (monocarboxylic acid transporter 7); similar to solute carrier family 16, member 6 | 12.8 | | 15.6 | | **APCS** | | | | amyloid P component, serum | | | -10.7 | | | 9.7 | |
| **CXCL11** | | chemokine (C-X-C motif) ligand 11 | 7.9 | | 2.8 | | **FOXS1** | | | | forkhead box S1 | | | -10.0 | | | 8.4 | |
| **BATF2** | | basic leucine zipper transcription factor, ATF-like 2 | 7.1 | | 1.1 | | **HSD17B6** | | | | hydroxysteroid (17-beta) dehydrogenase 6 homolog (mouse) | | | -9.6 | | | 11.1 | |
| **IFIT2** | | interferon-induced protein with tetratricopeptide repeats 2 | 6.7 | | 4.0 | | **RASSF2** | | | | Ras association (RalGDS/AF-6) domain family member 2 | | | -9.4 | | | 7.4 | |
| **RSAD2** | | radical S-adenosyl methionine domain containing 2 | 6.7 | | 0.1 | | **CHRDL2** | | | | chordin-like 2 | | | -8.9 | | | 7.5 | |
| **IFIT1B** | | interferon-induced protein with tetratricopeptide repeats 1-like | 6.4 | | 0.7 | | **ACTG2** | | | | actin, gamma 2, smooth muscle, enteric | | | -8.4 | | | 6.1 | |
| **FCRLA** | | Fc receptor-like A | 5.9 | | 3.8 | | **H19** | | | | H19, imprinted maternally expressed transcript (non-protein coding) | | | -7.7 | | | 3.1 | |
| **SGCG** | | sarcoglycan, gamma (35kDa dystrophin-associated glycoprotein) | 5.8 | | 1.1 | | **MXRA5** | | | | matrix-remodelling associated 5 | | | -7.5 | | | 4.4 | |
| **CMPK2** | | cytidine monophosphate (UMP-CMP) kinase 2, mitochondrial | 5.8 | | 0.4 | | **HP** | | | | haptoglobin-related protein; haptoglobin | | | -7.1 | | | 2.4 | |
| **TGFβ3 Group** | | | | | | | | | | | | | | | | | | |
| **Total number of genes regulated (cutoff 1.5):** | | | | | | | | | | | | 4007 | | | | | | |
| **Top 10 upregulated genes** | | | | | | | **Top 10 downregulated genes** | | | | | | | | | | | |
| **Gene** | **Gene name** | | | **mean fold induction** | **SD** | | **Gene** | | | **Gene name** | | | **mean fold induction** | | | **SD** | | |
| **AMIGO2** | adhesion molecule with Ig-like domain 2 | | | 147.1 | 44.5 | | **FIGF** | | | c-fos induced growth factor (vascular endothelial growth factor D) | | | -90.6 | | | 38.2 | | |
| **TSPAN2** | tetraspanin 2 | | | 125.1 | 63.1 | | **COLEC12** | | | collectin sub-family member 12 | | | -67.9 | | | 39.5 | | |
| **NOX4** | NADPH oxidase 4 | | | 114.5 | 51.8 | | **STEAP4** | | | STEAP family member 4 | | | -67.7 | | | 21.9 | | |
| **COL10A1** | collagen, type X, alpha 1 | | | 101.4 | 11.3 | | **KIT** | | | similar to Mast/stem cell growth factor receptor precursor (SCFR) (Proto-oncogene tyrosine-protein kinase Kit) (c-kit) (CD117 antigen); v-kit Hardy-Zuckerman 4 feline sarcoma viral oncogene homolog | | | -66.8 | | | 32.2 | | |
| **COL4A4** | collagen, type IV, alpha 4 | | | 67.5 | 46.3 | | **EGFL6** | | | EGF-like-domain, multiple 6 | | | -62.7 | | | 37.6 | | |
| **NPTX1** | neuronal pentraxin I | | | 64.2 | 22.0 | | **ASPA** | | | aspartoacylase (Canavan disease) | | | -59.7 | | | 44.1 | | |
| **C18orf1** | chromosome 18 open reading frame 1 | | | 63.3 | 1.6 | | **ARHGAP6** | | | Rho GTPase activating protein 6 | | | -50.3 | | | 21.9 | | |
| **PCDH19** | protocadherin 19 | | | 59.3 | 17.3 | | **S1PR1** | | | sphingosine-1-phosphate receptor 1 | | | -46.7 | | | 1.8 | | |
| **ST6GAL2** | ST6 beta-galactosamide alpha-2,6-sialyltranferase 2 | | | 56.4 | 21.4 | | **DCLK1** | | | doublecortin-like kinase 1 | | | -38.7 | | | 22.6 | | |
| **SLC22A2** | solute carrier family 22 (organic cation transporter), member 2 | | | 47.3 | 17.7 | | **EMCN** | | | endomucin | | | -36.5 | | | 4.4 | | |

**Supplemental Table 4.**

| **Official Gene Symbol** | **Name** | **CD24 Up** | **CD24 Up** | **CD24 Down** | **CD24 Down** |
| --- | --- | --- | --- | --- | --- |
| A2M | alpha-2-macroglobulin | 1.8 | 1.5 | -2.3 | -2.0 |
| ACTA2 | actin, alpha 2, smooth muscle, aorta | 1.8 | 1.8 | -4.9 | -1.8 |
| ACTG2 | actin, gamma 2, smooth muscle, enteric | 3.7 | 4.0 | -12.7 | -4.0 |
| AEBP1 | AE binding protein 1 | 1.5 | 1.8 | -2.5 | -1.9 |
| ALPL | alkaline phosphatase, liver/bone/kidney | 1.6 | 2.1 | -2.8 | -2.1 |
| APOE | hypothetical LOC100129500; apolipoprotein E | 2.3 | 2.7 | -4.0 | -2.2 |
| C20orf103 | chromosome 20 open reading frame 103 | 2.7 | 2.3 | -2.1 | -3.3 |
| CHRDL2 | chordin-like 2 | 2.5 | 2.4 | -14.2 | -3.6 |
| CNN1 | calponin 1, basic, smooth muscle | 2.3 | 2.2 | -11.6 | -2.2 |
| COL14A1 | collagen, type XIV, alpha 1 | 1.6 | 1.6 | -4.8 | -1.7 |
| COL18A1 | collagen, type XVIII, alpha 1 | 2.4 | 2.3 | -1.5 | -2.0 |
| COL1A1 | collagen, type I, alpha 1 | 2.6 | 2.6 | -6.5 | -3.1 |
| EPGN | epithelial mitogen homolog (mouse) | -1.7 | -1.7 | 7.9 | 2.5 |
| FBLN2 | fibulin 2 | 1.9 | 1.9 | -2.4 | -2.6 |
| FMO2 | flavin containing monooxygenase 2 (non-functional) | 2.7 | 2.1 | -12.3 | -1.5 |
| FOXS1 | forkhead box S1 | 16.9 | 8.3 | -15.9 | -4.0 |
| GDNF | glial cell derived neurotrophic factor | -1.8 | -2.3 | 1.8 | 1.8 |
| GGT5 | gamma-glutamyltransferase 5 | 2.2 | 2.2 | -2.4 | -1.6 |
| GPM6B | glycoprotein M6B | 2.4 | 2.1 | -2.1 | -2.4 |
| HAPLN1 | hyaluronan and proteoglycan link protein 1 | 1.7 | 1.9 | -5.5 | -1.6 |
| HAS3 | hyaluronan synthase 3 | 1.5 | 1.7 | -7.0 | -3.2 |
| HERC5 | hect domain and RLD 5 | -2.2 | -2.5 | 5.4 | 4.5 |
| HEY2 | hypothetical LOC100129733; hairy/enhancer-of-split related with YRPW motif 2 | 2.2 | 1.9 | -3.0 | -3.3 |
| HMOX1 | heme oxygenase (decycling) 1 | -1.7 | -1.6 | 3.8 | 2.2 |
| IDO1 | indoleamine 2,3-dioxygenase 1 | -2.2 | -2.4 | 5.2 | 4.1 |
| IGFBP2 | insulin-like growth factor binding protein 2, 36kDa | 1.5 | 1.7 | -3.5 | -1.5 |
| IGFBP5 | insulin-like growth factor binding protein 5 | 2.8 | 2.2 | -2.2 | -2.0 |
| JAK3 | Janus kinase 3 | 2.1 | 1.5 | -2.9 | -1.9 |
| KIF20A | kinesin family member 20A | 1.5 | 1.8 | -1.8 | -2.0 |
| LOC100126784 | hypothetical LOC100126784 | -1.5 | -2.4 | 1.6 | 2.0 |
| MFAP5 | microfibrillar associated protein 5 | 1.8 | 1.7 | -4.4 | -2.0 |
| MLC1 | megalencephalic leukoencephalopathy with subcortical cysts 1 | 2.5 | 2.1 | -3.9 | -3.4 |
| MMP1 | matrix metallopeptidase 1 (interstitial collagenase) | -1.6 | -1.7 | 2.7 | 1.5 |
| MRVI1 | murine retrovirus integration site 1 homolog | 2.4 | 1.9 | -5.3 | -1.9 |
| MSR1 | macrophage scavenger receptor 1 | -1.6 | -1.7 | 1.5 | 2.4 |
| NAMPT | nicotinamide phosphoribosyltransferase | -1.9 | -1.5 | 2.1 | 1.6 |
| P2RY6 | pyrimidinergic receptor P2Y, G-protein coupled, 6 | 1.6 | 1.6 | -2.7 | -1.7 |
| POM121L9P | POM121 membrane glycoprotein-like 9 (rat) pseudogene | 2.7 | 2.7 | -2.5 | -1.8 |
| PPIL6 | peptidylprolyl isomerase (cyclophilin)-like 6 | -2.2 | -1.5 | 1.6 | 2.1 |
| PTPN22 | protein tyrosine phosphatase, non-receptor type 22 (lymphoid) | -1.6 | -1.9 | 2.7 | 2.3 |
| SERPINA3 | serpin peptidase inhibitor, clade A (alpha-1 antiproteinase, antitrypsin), member 3 | 3.7 | 4.1 | -2.2 | -2.5 |
| SLC16A6 | solute carrier family 16, member 6 (monocarboxylic acid transporter 7); similar to solute carrier family 16, member 6 | -2.1 | -2.3 | 23.8 | 1.7 |
| SOD2 | superoxide dismutase 2, mitochondrial | -1.6 | -1.7 | 1.8 | 1.9 |
| SORCS2 | sortilin-related VPS10 domain containing receptor 2 | 1.9 | 1.9 | -3.1 | -2.0 |
| SUSD2 | sushi domain containing 2 | 3.3 | 3.1 | -6.0 | -1.6 |
| THY1 | Thy-1 cell surface antigen | 1.7 | 1.9 | -2.1 | -2.2 |
| TREM2 | triggering receptor expressed on myeloid cells 2 | 1.7 | 1.7 | -6.3 | -1.8 |
| TRIM14 | tripartite motif-containing 14 | -1.6 | -1.6 | 1.6 | 1.5 |
| UHRF1 | ubiquitin-like with PHD and ring finger domains 1 | 1.6 | 1.6 | -2.0 | -2.0 |
| WFDC1 | WAP four-disulfide core domain 1 | 1.8 | 2.8 | -3.8 | -2.0 |
| ZNF469 | zinc finger protein 469 | 2.0 | 1.8 | -1.8 | -1.6 |

Supplemental Table 5.

| **Canonical Pathway** | **-log(p-value)** | | |
| --- | --- | --- | --- |
|  | **CD24 Down** | **CD24 Up** | **TGFb3** |
| **Hepatic Fibrosis / Activation of Hepatic Stellate Cells** | 13.20 | 9.98 | 15.45 |
| **Atherosclerosis Signaling** | 8.55 | 6.29 | 6.74 |
| **Acute Phase Response Signaling** | 5.33 | 2.31 | 5.60 |
| **Granulocyte Adhesion and Diapedesis** | 8.21 | 5.86 | 4.54 |
| **LXR/RXR Activation** | 4.77 | 3.00 | 7.85 |
| **Role of Macrophages, Fibroblasts and Endothelial Cells in Rheumatoid Arthritis** | 3.31 | 1.75 | 6.05 |
| **Agranulocyte Adhesion and Diapedesis** | 8.68 | 6.27 | 3.59 |
| **Role of Osteoblasts, Osteoclasts and Chondrocytes in Rheumatoid Arthritis** | 3.57 | 3.16 | 4.11 |
| **Inhibition of Matrixmetalloproteinases** | 6.14 | 2.00 | 4.17 |

Supplemental Table 6.

|  | **CD24 Up Group** | | **CD24 Down Group** | | **TGFβ3 Group** | |
| --- | --- | --- | --- | --- | --- | --- |
| **Gene** | **fold increase relative to control** | **SD** | **fold increase relative to control** | **SD** | **fold increase relative to control** | **SD** |
| **FOXS1** | 12.6 | 6.1 | -10.0 | 8.4 | 20.4 | 2.7 |
| **ACTA2** | 1.8 | 0.0 | -3.4 | 2.2 | 3.1 | 1.4 |
| **COL1A1** | 2.6 | 0.0 | -4.8 | 2.4 | 6.6 | 3.6 |
| **CNN1** | 2.3 | 0.1 | -6.9 | 6.6 | 7.8 | 5.1 |

**Supplemental Figure 1.**

**
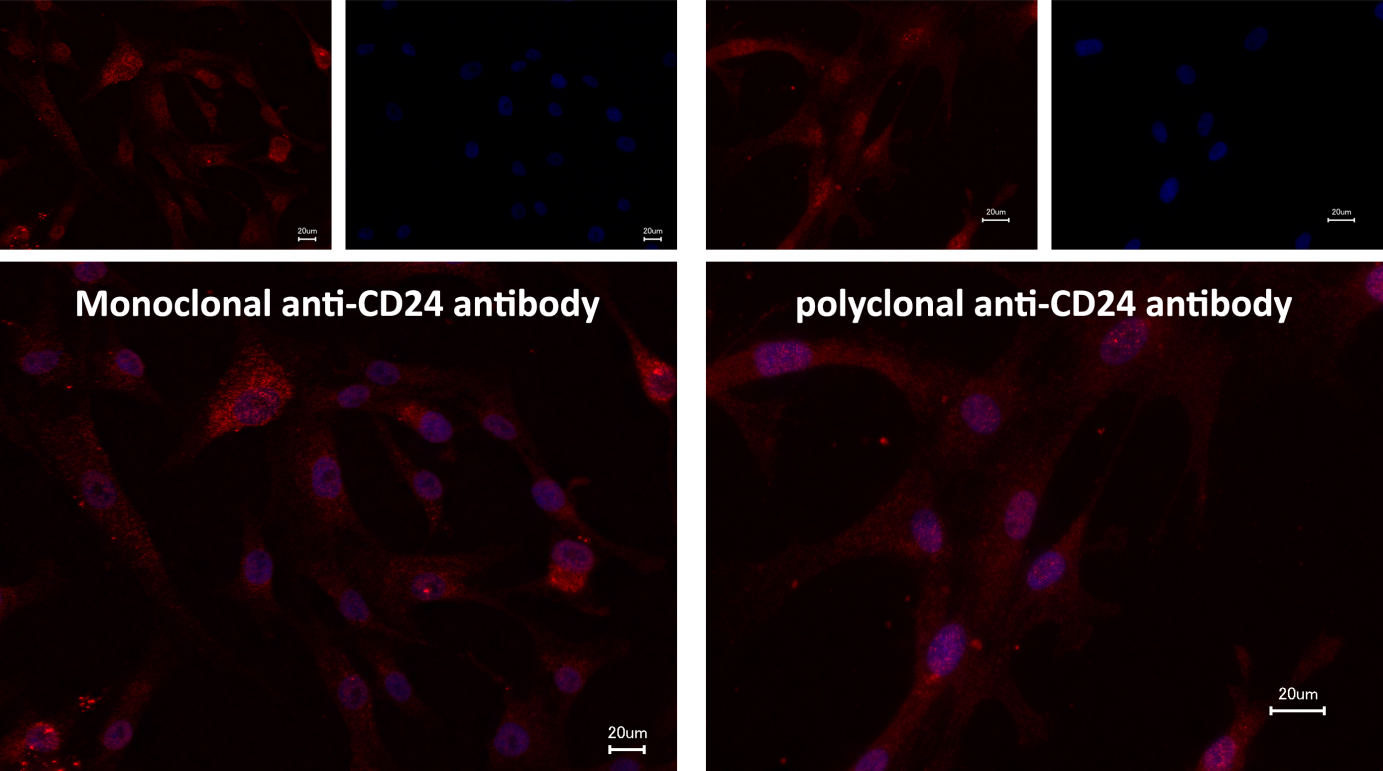
**

**Supplemental Figure 2.**

**
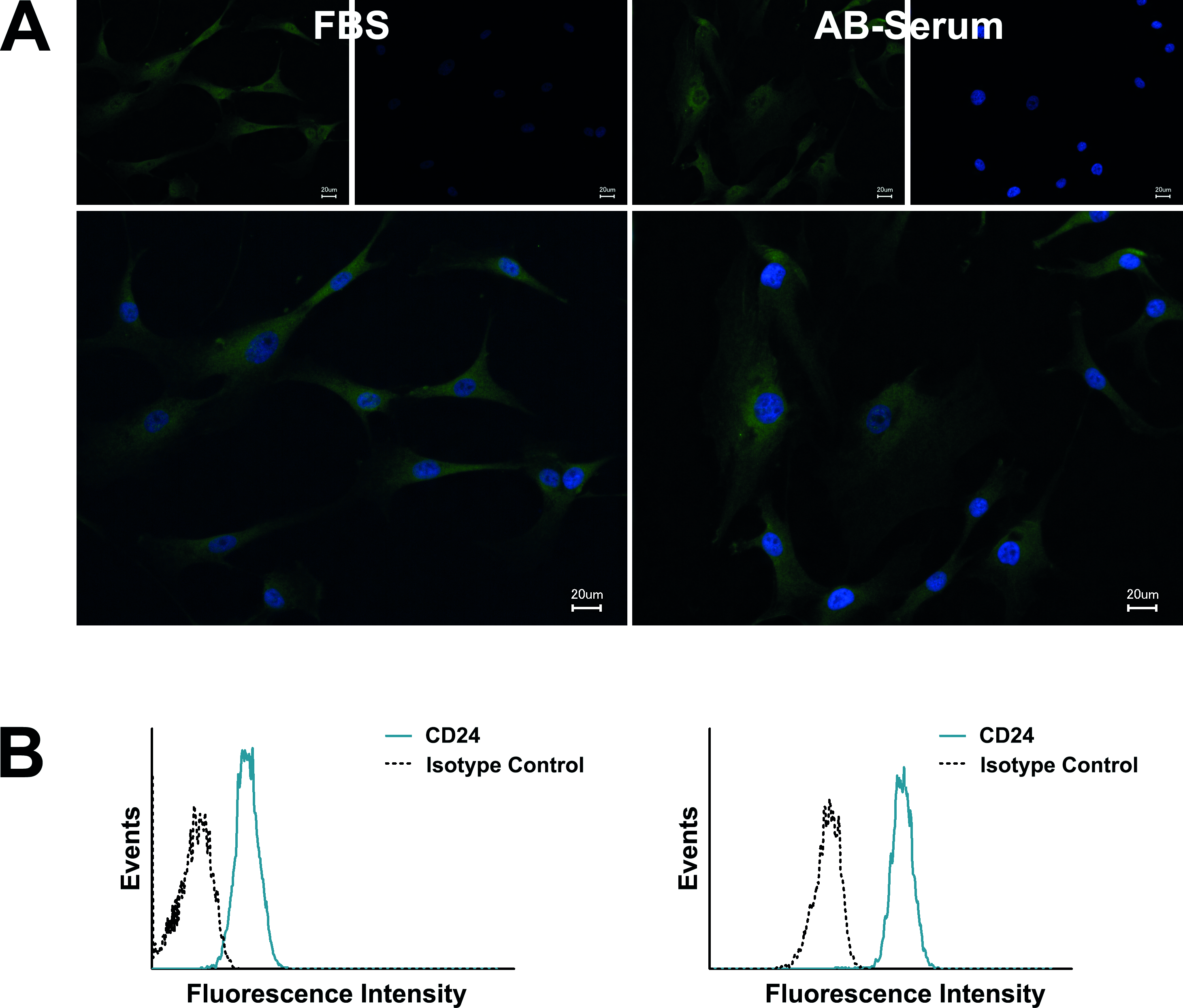
**

**Supplemental Figure 3.**

**
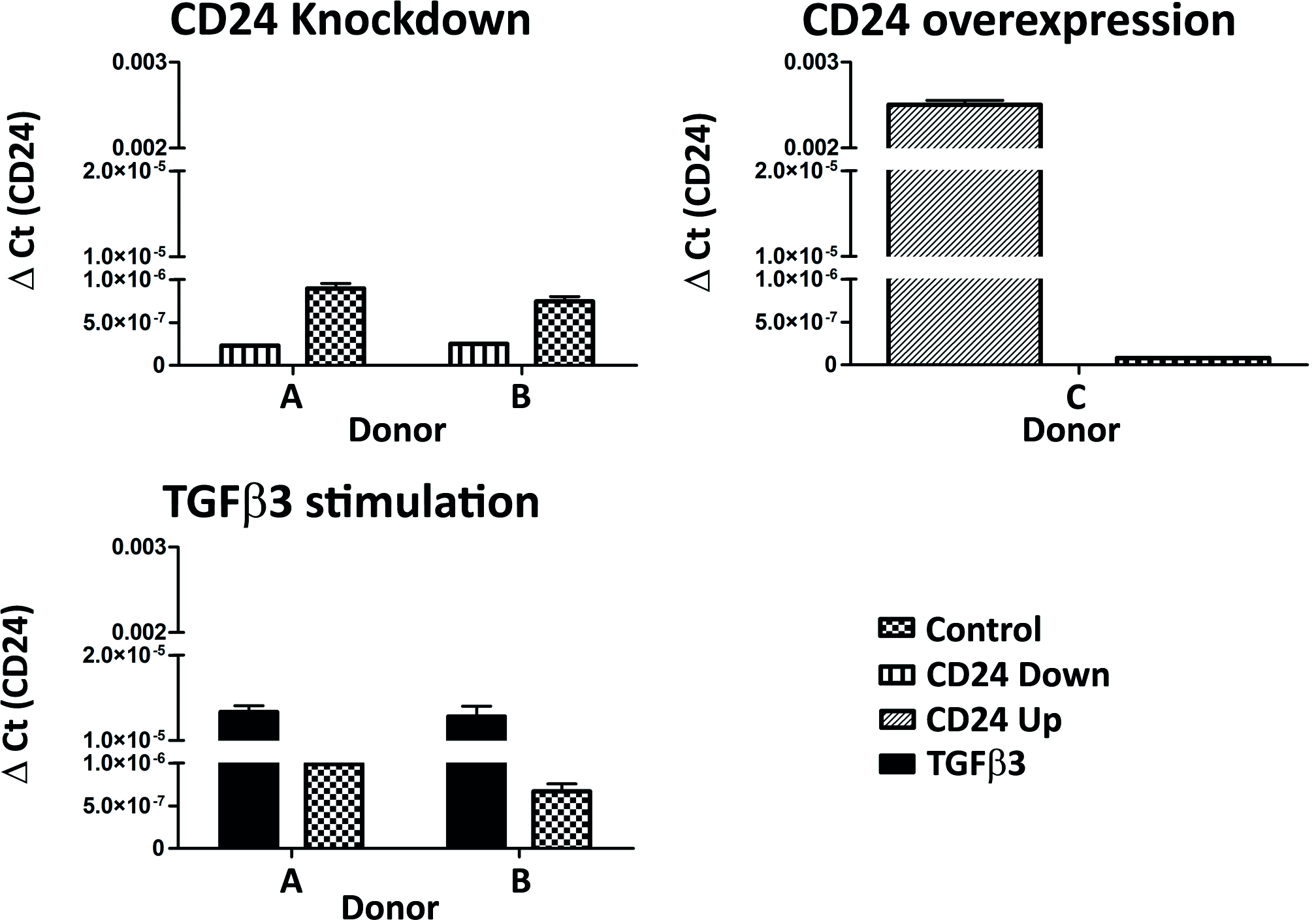
**

**Supplemental Figure 4.**

**
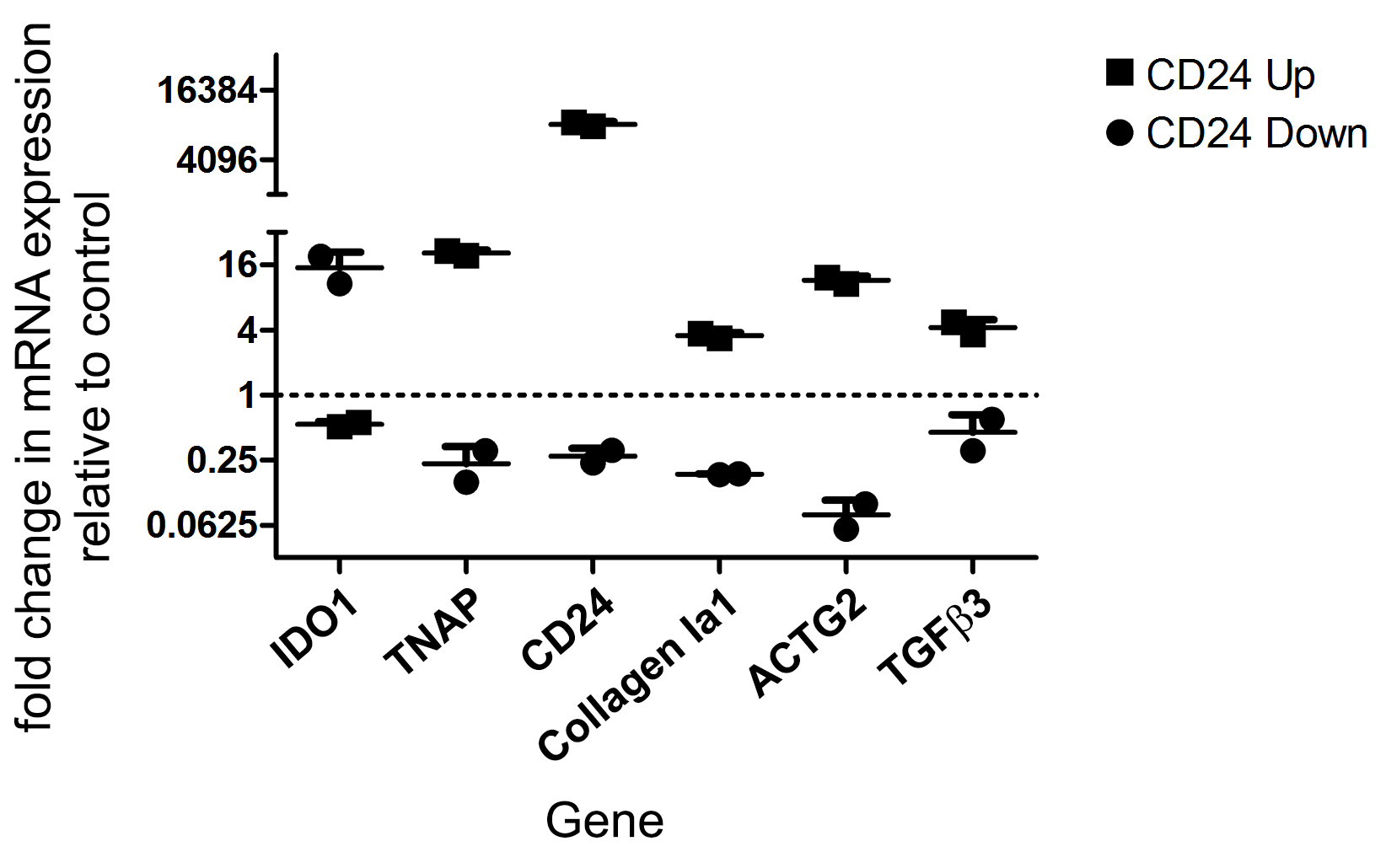
**

**Supplemental Figure 5.
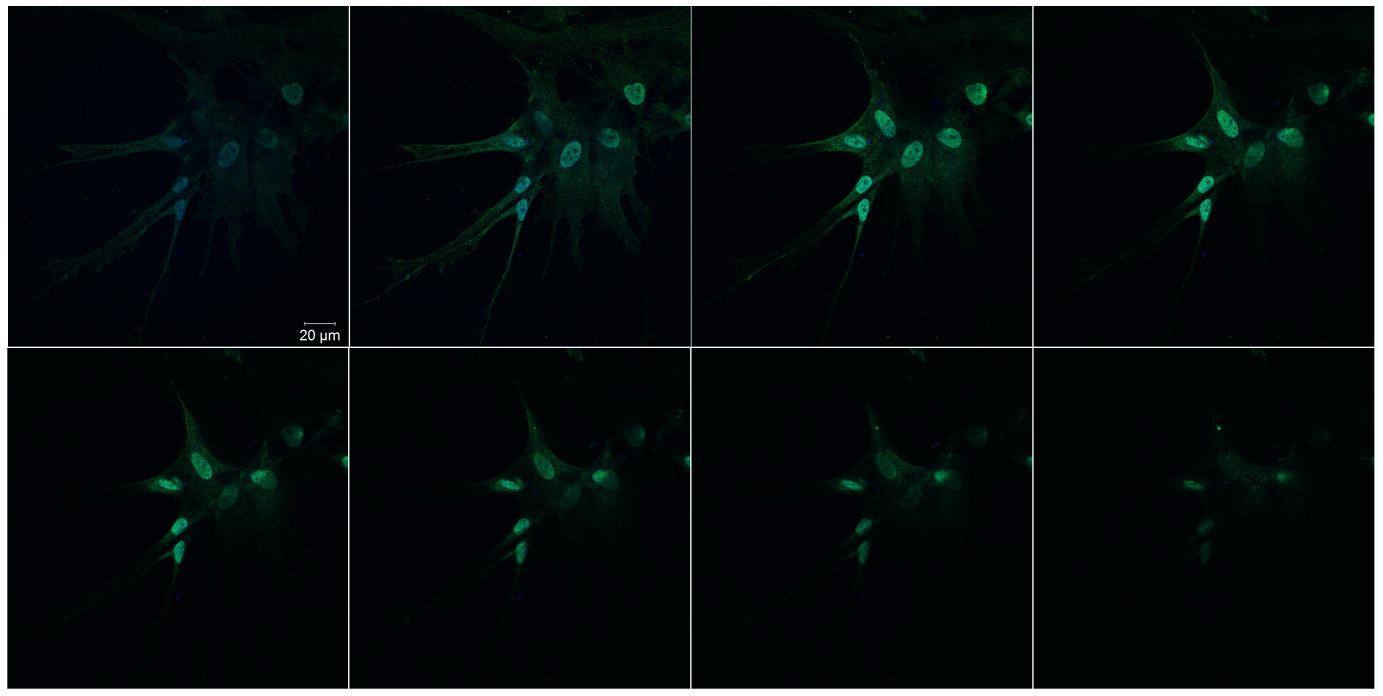
**
